# The Rac1 Inhibitor NSC23766 Exerts Anti-Influenza Virus Properties by Affecting the Viral Polymerase Complex Activity

**DOI:** 10.1371/journal.pone.0088520

**Published:** 2014-02-11

**Authors:** Rüdiger Dierkes, Kathrin Warnking, Swantje Liedmann, Roman Seyer, Stephan Ludwig, Christina Ehrhardt

**Affiliations:** Institute of Molecular Virology (IMV), Centre of Molecular Virology (ZMBE), Westfälische Wilhelms-University, Münster, Germany; Kliniken der Stadt Köln gGmbH, Germany

## Abstract

The frequent emergence of new influenza viruses in the human population underlines the urgent need for antiviral therapeutics in addition to the preventative vaccination against the seasonal flu. To circumvent the development of resistance, recent antiviral approaches target cellular proteins needed by the virus for efficient replication. We investigated the contribution of the small GTPase Rac1 to the replication of influenza viruses. Inhibition of Rac1 by NSC23766 resulted in impaired replication of a wide variety of influenza viruses, including a human virus strain of the pandemic from 2009 as well as highly pathogenic avian virus strains. Furthermore, we identified a crucial role of Rac1 for the activity of the viral polymerase complex. The antiviral potential of NSC23766 was confirmed in mouse experiments, identifying Rac1 as a new cellular target for therapeutic treatment of influenza virus infections.

## Introduction

Influenza viruses (IVs) are family members of *Orthomyxoviridae* and are grouped into three different subtypes (A, B and C). Among those, subtype A viruses are the major cause of seasonal outbreaks, affecting the elderly and immune compromised persons but also bear the potential to cause pandemics. The emergence of the pandemic swine-origin IV in 2009, the recent human infections with avian H7N9 viruses, and the ongoing human infections with highly pathogenic avian H5N1 viruses highlight the permanent threat elicited by these pathogens.

Although vaccination is an efficient prevention of IV infection, this approach may fail in case of wrong predictions for the annual vaccines or in a pandemic situation when availability of the vaccine is insufficient. Accordingly, other antiviral strategies to control infections are required. Currently, the commonly available drugs target either the viral ion channel M2 (amantadine, rimantadine) or the viral neuraminidase (NA; oseltamivir, zanamivir). Unfortunately, usage of these drugs results in the frequent development of resistant virus variants. Therefore, clinical use of M2 ion channel blockers is no longer recommended [1]. In recent years, novel antiviral approaches have been directed against cellular factors, which are essential for viral replication [2], [3]. Such alternative strategies seem to offer a higher barrier for the development of drug resistance.

Rac1 belongs to the family of Rho GTPases that regulate a wide variety of cellular processes, such as cytoskeleton organization, gene expression, cell cycle progression, and cell motility [4]. To maintain their regulatory functions, these molecules cycle between a GTP-bound (active) state and a GDP-bound (inactive) state. The turnover from active to inactive state is catalyzed by its intrinsic GTPase activity. The cycle is tightly regulated by two classes of proteins: activating guanine nucleotide exchange factors (GEFs), which catalyze the exchange of GDP to GTP, and GTPase-activating proteins (GAPs), which stimulate the hydrolysis of the bound GTP leading to inactivation of Rac1.

The key role of Rac1-dependent signaling in important cellular functions led to the hypothesis that it might be essential for the replication of different viruses as well. Indeed, a growing number of reports describe a significant impact of Rac1 on the life cycle of diverse viruses. Among those, virus-supportive as well as virus-suppressive functions have been identified. Rac1 activity is needed for the internalization of human immunodeficiency virus, vaccinia virus, and African swine fever virus [5]–[7]. Furthermore, vesicular trafficking of entering viral particles is influenced by Rac1 during infections with adenovirus, african swine fever virus, and Ebola virus [8]–[10]. In case of dengue viruses, Rac1 activity seems to impair the entry process and is downregulated during the early stages of the infection [11]. However, the same report suggests a virus-supportive role of the GTPase during assembly and budding of dengue viruses.

In case of IV infections, we have shown that Rac1 is activated upon infection [12]. The over-expression of a dominant negative mutant form of Rac1 led to reduced interferon-β production, which is the main response of the innate immune system to IV infections. Consequently, an antiviral effect of Rac1 activity was proposed. However, besides this antiviral property of Rac1, we could not rule out a virus-supportive function during ongoing IV replication. Furthermore, it became apparent that several enzymes fulfill virus-supportive roles as well as antiviral functions within the IV life-cycle [13]. In the meantime, new tools to investigate the role of Rac1 had become available. Gao et al. identified the small chemical compound NSC23766 as a Rac1-inhibiting drug and showed that it interferes with a binding-groove of Rac1, a domain that is involved in the determination of Rac1's specificity to certain GEFs [14], [15]. NSC23766 specifically inhibits Rac1 activity without effecting the closely related Rho-GTPases Cdc42 and RhoA. It blocks the interaction of Rac1 with its GEFs Tiam1 and Trio, without targeting the activation by other GEFs, such as Vav, Lbc or intersectin [15]. This high specificity for Rac1 and a small subset of GEFs suggests that NSC23766 is an ideal candidate to target specific Rac1-mediated signaling processes. While a recent study tested the effect of NSC23766 treatment on IV entry and ruled out an involvement of Rac1 in endocytosis of these viruses [16], we aimed to investigate the impact of NSC23766 treatment on IV replication.

## Materials and Methods

### Cells, Viruses and Infection Conditions

All experiments were performed in human lung epithelial cells (A549) grown in DMEM supplemented with 10% FBS. MDCKII cells were cultivated in MEM supplemented with 10% FBS and were used for propagation of the different influenza virus strains and for standard plaque assays. The infection procedure was performed as described previously [17]. The human recombinant influenza A virus strain A/Puerto-Rico/8/34 (H1N1) was generated using the pHW2000-based reverse genetic system (a kind gift from Dr. Robert G. Webster, Memphis, TN, USA) [18]. The swine-origin A/Hamburg/04/2009 (H1N1v) was obtained from Brunhilde Schweiger, German National Reference Centre for Influenza, Berlin. The highly pathogenic avian influenza A virus strain A/FPV/Bratislava/79 (H7N7) was taken from the virus strain collection of the Institute of Virology, Giessen, Germany. The A/Thailand/1(KAN-1)/2004 (H5N1) was isolated at the Siriraj Hospital, Mahidol University, Bangkok, Thailand and the human influenza B virus strain B/Maryland/59 was obtained from Thorsten Wolff, Robert-Koch-Institute, Berlin, Germany. Viral titers of cell culture supernatants and mouse lung lysates were determined by standard plaque assays. All experiments with the highly pathogenic avian influenza strains A/FPV/Bratislava/79 and A/Thailand/1(KAN-1)/2004 were conducted under BSL-3 conditions.

### Chemical Inhibitors

Rac1 inhibitor NSC23766 (N6-[2-[[4-(diethylamino)-1-methylbutyl]amino]-6-methyl-4-pyrimidinyl]-2-methyl-4,6-quinolinediamine trihydrochloride) (Tocris) was dissolved in H_2_O at a stock concentration of 50 mM. Unless otherwise indicated, it was used at a final concentration of 100 µM in cell culture experiments.

The M2 ion channel blocker amantadine (Sigma) was dissolved in H_2_O at a concentration of 2.5 mM and diluted to a concentration of 5 µM in cell culture medium. To inhibit protein translation, cycloheximide (Sigma) was used at a final concentration of 10 µg/ml. Staurosporine (Sigma) served as a positive control for induction of apoptosis (1 µM).

### Preparation of Nuclear Extracts, Western Blot Analysis, and Antibodies

To differentiate between cytosolic and nuclear localization of proteins, nuclear extracts were prepared. Therefore, 6×10^6^ A549 cells were treated as indicated, washed with PBS, and collected in 1 ml PBS. After centrifugation (5 min at 650 g and 4°C), cells were resuspended in 1 ml Roeder A buffer (10 mM HEPES pH 7.9, 1.5 mM MgCl_2_, 10 mM KCl supplemented with 0.5 mM DTT, 1 mM sodium vanadate, 5 mM benzamidine, 0.2 mM pefablock, 5 µg/ml leupeptin, and 5 µg/ml aprotinin) and incubated on ice for ten minutes. After addition of Igepal CA-630 (Sigma) to a final concentration of 0.6%, cells were mixed for 30 s and incubated for another ten minutes on ice. Nuclei were pelleted by centrifugation (10 min at 2650 g and 4°C) and the supernatant (cytosolic fraction) was transferred to another tube. The pellet was washed with 1 ml Roeder A buffer and subsequently resuspended in 250 µl Roeder C buffer (25% (v/v) glycerol, 0.3 M NaCl, 1.5 mM MgCl_2_, 20 mM HEPES pH 7.9 supplemented with 0.5 mM DTT, 1 mM sodium vanadate, 5 mM benzamidine, 0.2 mM pefablock, 5 µg/ml leupeptin, and 5 µg/ml aprotinin). After incubation at 4°C over-night in an overhead shaker, the nuclear fraction was clarified by centrifugation (30 min at 21000 g and 4°C). Protein concentrations of cytosolic and nuclear fractions were determined using Bio-Rad Protein Assay (Bio-Rad). Subsequently the samples were treated as other protein lysates for Western blot analysis.

To analyze the expression of specific proteins, cell lysates were prepared as described previously [17] and total protein concentrations were determined with the BCA Protein Assay Kit (Pierce). After SDS-gel-electrophoresis and Western blotting, protein bands were visualized with an enhanced chemiluminescence reaction and imaged by a CCD camera-based system (Stella, raytest).

To evaluate the onset of apoptosis, PARP and its cleaved form were detected by an anti-PARP mouse mAb (BD). The viral non-structural protein 1 (NS1) was analyzed by an anti-NS1 mouse mAb (clone NS1-23-1; IMV Münster, Germany). The viral matrix protein 1 (M1) was detected by anti-M1 mouse mAb (Serotec). Expression of the viral polymerase-complex protein basic 1 (PB1) was monitored by an anti-PB1 (vK-20) goat pAb (Santa Cruz). Knockdown efficiency of Rac1 and Tiam1 was shown by specific antibodies for each protein (anti-Rac1 mouse mAb, Upstate; anti-Tiam1 rabbit pAb, abcam). As markers for cytosolic and nuclear fractions anti-α-Tubulin mouse mAb (Sigma) and anti-Drosha rabbit mAb (Cell Signaling) were used, respectively. To control equal protein loading, an anti-ERK2 (C-14) rabbit pAb (Santa Cruz) was used.

### Transient Transfections, Plasmids, siRNAs, and Reporter Gene Assays

Transfection of plasmid DNA as well as siRNA was performed with Lipofectamine2000 (Invitrogen) according to the manufacturer's protocol. For knockdown of Rac1 and Tiam1, predesigned siRNAs (Qiagen) against Rac1 (Hs_Rac1_6) or Tiam1 (Hs_Tiam1_3), respectively, were used (30 pmol per 12-well). For negative control, non-silencing AllStars Negative Control siRNA (Qiagen) was employed.

To stimulate the interferon response 0.5 µg total RNA isolated from A549 cells infected with A/FPV/Bratislava/79 (moi = 5) for six hours were used for transfection. RNA of mock-infected A549 cells was transfected as control.

To assay general protein expression, 0.5 µg of a reporter gene plasmid encoding the firefly luciferase under control of a CMV promoter was transfected per 24-well. For analysis of viral polymerase activity, a mini-genome system was used [19]. The *Pol*I-driven reporter plasmid pHW72-luc, encoding the antisense-luciferase gene flanked by the viral RNA promoters, and a mixture of expression plasmids for the proteins of the viral polymerase complex (PB1, PB2, PA) and NP of influenza virus A/Puerto-Rico/8/34 (H1N1) based on the pHW2000 vector were transfected (0.3 µg of each plasmid per 24-well). Six hours after transfection cells were supplemented with fresh medium with or without Rac1 inhibitor NSC23766. After 24 h of incubation, luciferase activity was quantified in a luminometer according to standard procedures [20]. Measured relative light units were normalized to protein concentrations determined with the Bio-Rad Protein Assay (Bio-Rad).

### Analysis of Cellular Metabolic Activity, Cytostasis, and Apoptosis

The metabolic activity of inhibitor-treated A549 cells was analyzed by MTT assay as described previously [21]. Cytostatic effects of NSC23766 were analyzed using cell proliferation reagent WST-1 (Roche) according to manufacturer's protocol. The amount of apoptotic cells was determined by flow cytometric analysis of propidium iodide-stained cells according to a protocol by Riccardi and Nicoletti [22].

### Ethics Statement

All animal studies were performed in accordance with the German regulations of the Society for Laboratory Animal Science (GVSOLAS) and the European Health Law of the Federation of Laboratory Animal Science Associations (FELASA). The protocol was approved by the Landesamt für Natur, Umwelt und Verbraucherschutz Nordrhein-Westfalen (LANUV-NRW), Germany (permit Az 8.87-50.10.36.09.007 and Az. 84-02.04.2013.A232).

### Mouse Experiments

In mouse experiments female and male BALB/c mice (10-17 weeks) were used. All data that are presented in the results section were obtained in two independent experiments with groups of 3-4 animals. For intranasal infection, BALB/c mice were anesthetized by intraperitoneal injection of ketamine (Ceva) and xylazin (Ceva). Indicated amounts of virus were diluted in 50 µl PBS and 25 µl were applied in each nostril.

Infected mice were treated twice per day for 20 min in an inhalation chamber. During each run, approximately 3 ml of an aqueous solution of NSC23766 (0.1 or 1 mg/ml) or water were nebulized. With this experimental setup, an estimated amount of 0.3 to 1% of the solution is delivered to the lung [23], leading to a maximum dosage of 3 mg/kg body weight/day.

To determine the virus load in the lung, mice were sacrificed by cervical dislocation following anesthesia with isoflurane (AbbVie) three days post-infection (p.i.) and lungs were homogenized at equal ratios (w/v) in PBS using a FastPrep-24 homogenizer (MP Biomedicals) with Lysing Matrix D (MP Biomedicals). The samples were centrifuged at 10000 g for ten minutes, and the supernatants were taken for plaque assay.

To draw a Kaplan-Meier survival curve body weight and water uptake were monitored once per day for a total period of 16 days post inoculation. In addition, the general state of health was observed twice per day. At the end of an experiment or when mice lost 25% of their initial body weight they were humanely euthanized by cervical dislocation after anesthesia with isoflurane.

### Quantitative Real-Time PCR

Total RNA of cells was isolated with the RNeasy mini kit (Qiagen) according to the manufacturer's protocol. Reverse transcription of mRNA was done as described before [17]. The cDNA was used for qRT-PCR with Brilliant QPCR SYBR Green (Stratagene) according to manufacturer's manual. After 40 amplification cycles, relative RNA amounts were calculated by using the 2^−ΔΔCT^ method [24]. Primers: GAPDH_fwd *5′-GCA AAT TTC CAT GGC ACC GT-3′*, GAPDH_rev *5′-GCC CCA CTT GAT TTT GGA GG-3′*, NS_fwd *5′-GAG GAC TTG AAT GGA ATG ATA ACA-3′*, NS_rev *5′-GTC TCA ATT CTT CAA TCA ATC AAC CAT C-3′*, PB1_fwd *5′-CAT ACA GAA GAC CAG TCG GGA T-3′*, PB1_rev *5′-GTC TGA GCT CTT CAA TGG TGG A-3*′, MxA_fwd *5′-GTT TCC GAA GTG GAC ATC GCA-3′,* MxA_rev *5′-GAA GGG CAA CTC CTG ACA GT-3′*

## Results

### NSC23766 Inhibits Replication of Influenza A and B Viruses

**Figure 1:**
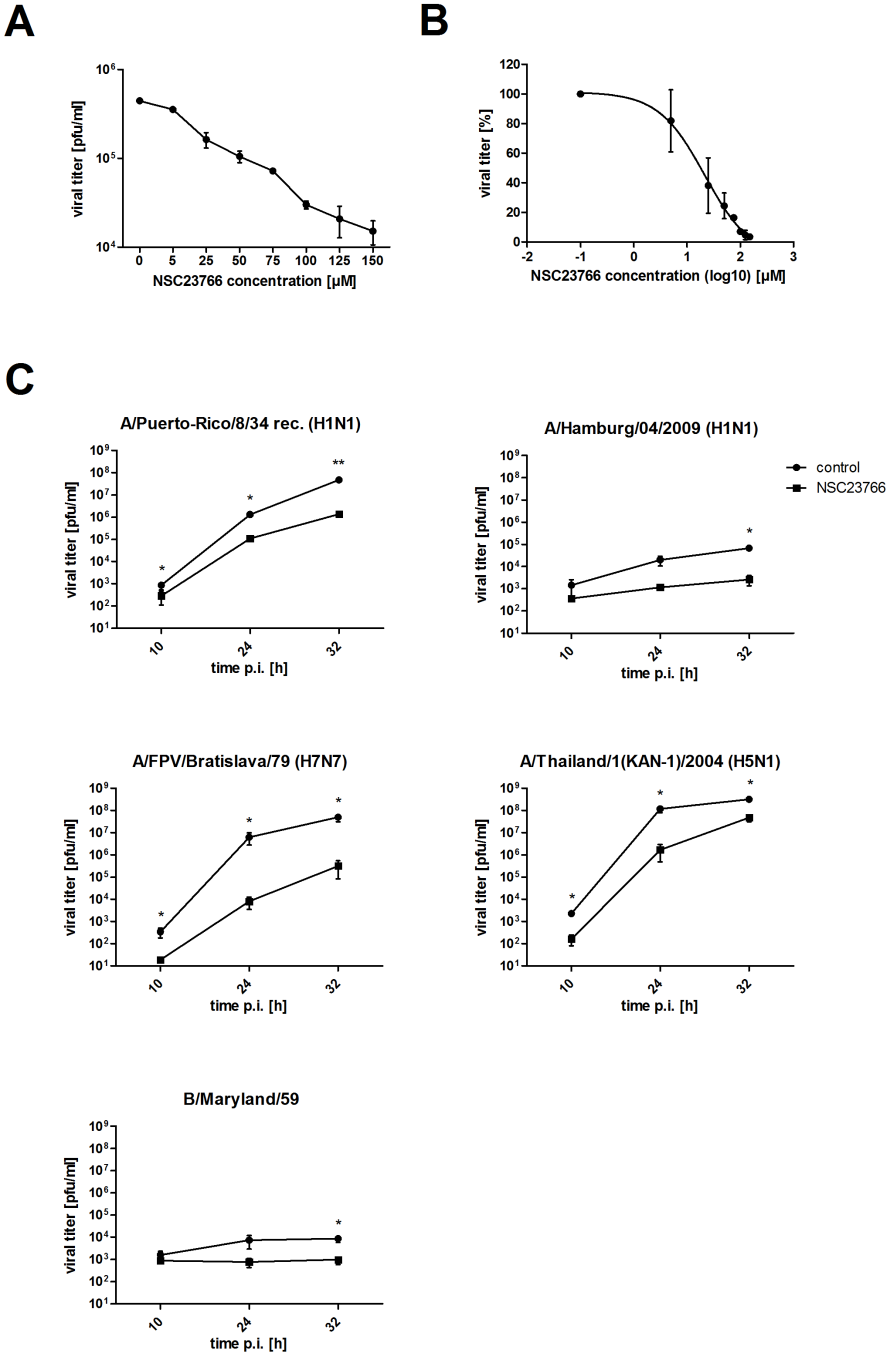
IV replication is impaired upon Rac1 inhibition by treatment with NSC23766. (A) A549 cells were infected with A/Puerto-Rico/8/34 rec. (moi = 0.01) for 30 min and subsequently treated with the indicated amounts of NSC23766 for 24 h. (B) Dose-response data depicted in A were used to determine the EC_50_ curve with GraphPad Prism 5 Software. (C) A549 cells were infected with A/Puerto-Rico/8/34 rec. (moi = 0.01), A/Hamburg/04/2009 (moi = 0.1), A/FPV/Bratislava/79, A/Thailand/1(KAN-1)/2004 (moi = 0.001), or B/Maryland/59 (moi = 0.1) and subsequently incubated with NSC23766 (100 µM) for 10, 24, or 32 h. (A, C) Progeny virus yields were determined by plaque assays. Data represent means ± SD of at least three independent experiments with two biological samples. Statistical significance was evaluated by Student's *t*-test (* p<0.05; ** p<0.01).

Initially we elucidated whether the specific inhibitor NSC23766, which prevents the interaction of Rac1 with its GEFs Tiam1 and Trio, exerts an antiviral effect against IV infection in cultured cells. A549 cells were infected with the recombinant human model IV A/Puerto-Rico/8/34 and treated with different concentrations of NSC23766 30 min p.i. for 24 h. Determination of progeny virus titers revealed a dose-dependent antiviral activity of the compound (Fig. 1A). The highest reduction of virus titers of about 90% was achieved at a concentration of 100 µM, while higher concentrations could not further enhance this antiviral effect. From a dose-response curve (Fig. 1B), an EC_50_ value of approximately 22 µM was calculated. NSC23766 did not only inhibit replication of A/Puerto-Rico/8/34, but also exhibited antiviral activity against several other IV subtypes (Fig. 1C). Determination of progeny virus titers of the swine-origin pandemic virus A/Hamburg/04/2009, the highly pathogenic avian isolate A/FPV/Bratislava/79, the human isolate of the avian A/Thailand/1(KAN-1)/2004, and B/Maryland/59 revealed a significant reduction upon NSC23766 treatment in the first (10 h) and following replication cycles (24 h, 32 h). These data indicate a broad antiviral activity of NSC23766 towards different IV strains, and a fundamental and highly conserved role of Rac1 during viral reproduction.

### NSC23766 Treatment Does Not Show Harmful Effects on Cell Health

**Figure 2:**
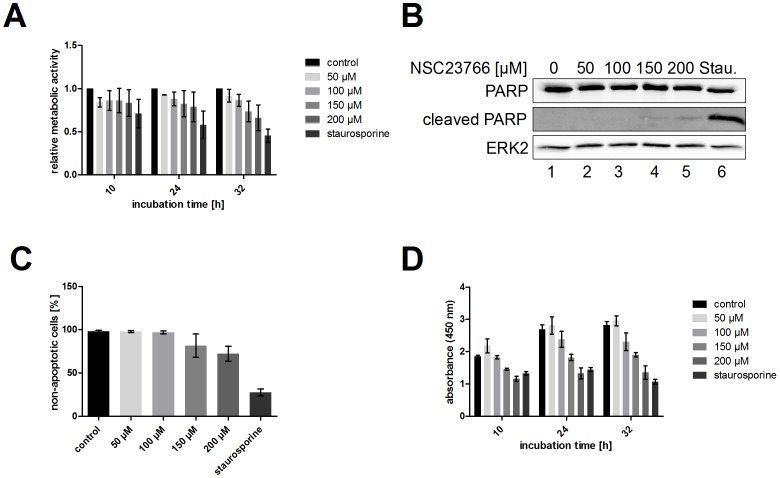
NSC23766 does not cause cytotoxic side-effects on A549 cells. A549 cells were treated with the indicated concentrations of NSC23766 or staurosporine (1 µM), which served as a positive control. After 10, 24, or 32 h incubation, metabolic activity was measured via MTT assay (A). The onset of apoptosis was analyzed after 24 h incubation either by the detection of PARP cleavage in Western blot analysis (B) or by propidium iodide staining (C). Cell proliferation was determined after indicated incubation times by usage of the cell proliferation reagent WST-1 detecting the absorbance at 450 nm (D). Data represent means ± SD of three independent experiments including four (A) or two (C) biological samples or means ± SD of three biological samples of one representative out of three conducted experiments (D).

Since the use of drugs targeting cellular factors may raise concerns about side effects on the host cell, we analyzed whether antiviral-acting concentrations of NSC23766 show harmful effects on cell health (Fig. 2). To investigate the impact of NSC23766 treatment on the metabolism of A549 cells, MTT assays were performed after 10, 24 and 32 h inhibitor treatment (Fig. 2A). The onset of apoptosis was evaluated after 24 h by detection of PARP cleavage in Western blot analysis (Fig. 2B) or measurement of DNA degradation via propidium iodide staining (Fig. 2C). Concentrations of up to 100 µM showed no severe effect on the metabolic activity (Fig. 2A) or apoptosis (Fig. 2B–C), while higher concentrations of 150 µM or 200 µM resulted in slightly reduced metabolic activity and enhanced apoptosis.

Since it is well known that Rac1 is involved in cell cycle progression, we tested also the cytostatic effect of NSC23766 on A549 cells at different concentrations (Fig. 2D). While concentrations of 150 and 200 µM led to a reduced cell proliferation, the concentration of 100 µM showed only a marginal cytostatic effect. There was no difference observable in cell proliferation of A549 cells treated with 50 µM NSC23766 and untreated cells.

In conclusion it can be stated that there are no adverse side-effects of NSC23766 on A549 cells at a concentration of 100 µM, which was used in further experiments.

### Rac1 Activity Is Required for Efficient IV Replication

**Figure 3:**
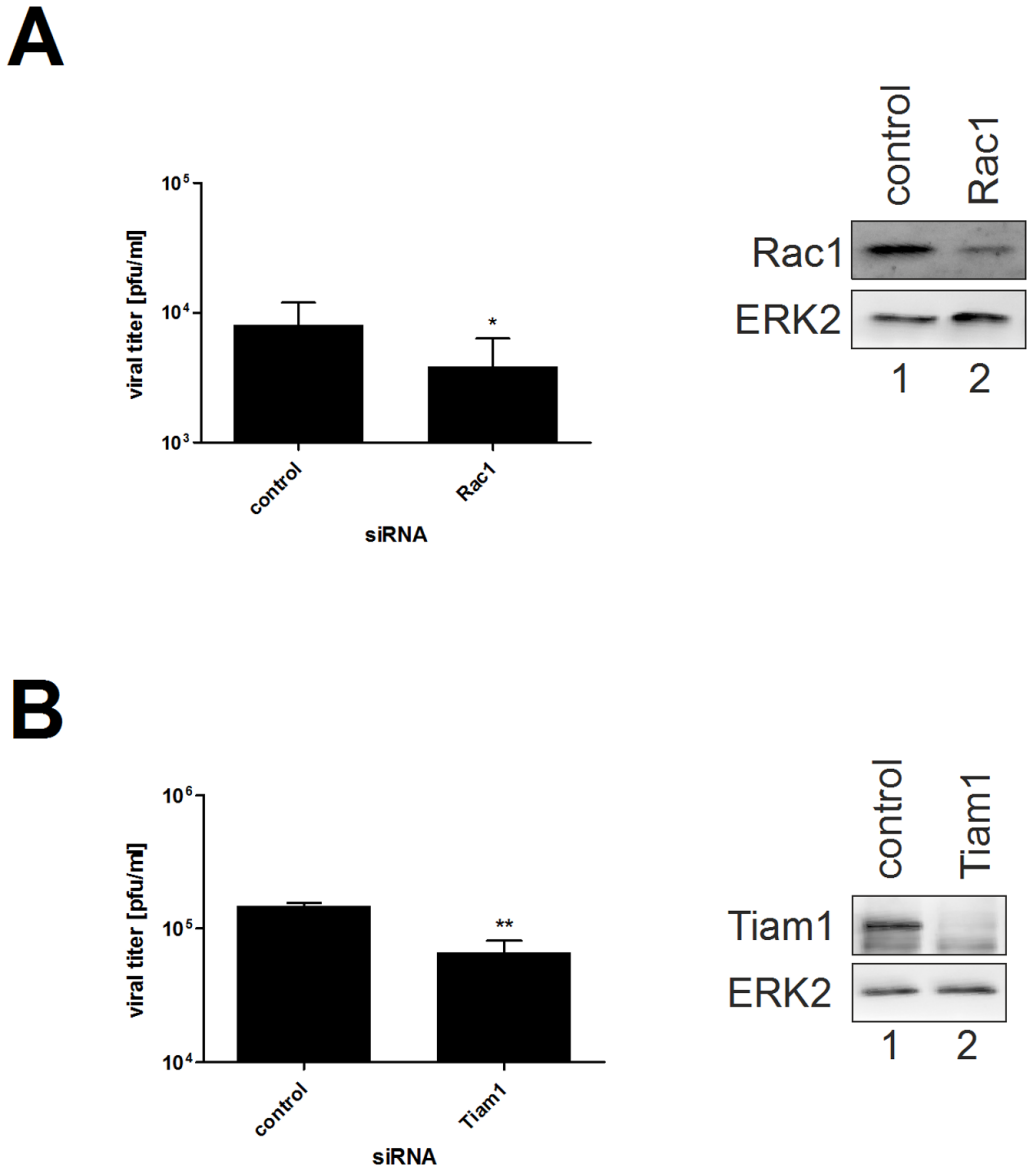
The siRNA mediated knockdown of Rac1 or Tiam1 leads to impaired viral replication. A549 cells were transfected with non-silencing control siRNA or siRNA against Rac1 (A) or Tiam1 (B) for 72 or 48 h, respectively. Thereafter, cells were infected with A/Puerto-Rico/8/34 rec. (moi = 0.01) for 24 h. Progeny virus yields were determined by plaque assays. Data represent means ± SD of three independent experiments including two biological samples. Statistical significance was evaluated by Student's *t*-test (* p<0.05; ** p<0.01). Efficient knockdown of Rac1 or Tiam1 was determined in Western blot analysis using specific antibodies against Rac1 or Tiam1, respectively. Detection of ERK2 served as a loading control.

As stated above, NSC23766 exerts inhibitory effects on Rac1 by preventing its interaction with the GEFs Tiam1 and Trio. To verify the role of Rac1 during IV replication, siRNAs against Rac1 or Tiam1 were used (Fig. 3). The knockdown of Rac1 was most efficient 72 h after transfection (∼80%), while efficient downregulation of Tiam1 was already achieved 48 h post transfection (∼86%). Silencing the expression of Rac1 led to significantly reduced replication of A/Puerto-Rico/8/34 24 h p.i. (Fig. 3A). Likewise, knockdown of the upstream factor Tiam1 led to reduced viral titers (Fig. 3B). Taken together, these data confirm a supportive role of Rac1 for IV replication and underline the specificity of the antiviral activity of NSC23766.

### Inhibition of Rac1 Signaling Leads to Reduced Viral Protein Synthesis

**Figure 4:**
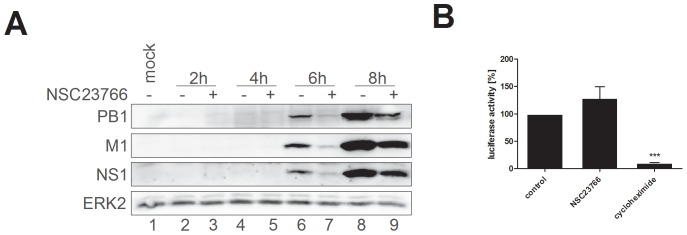
Inhibition of Rac1 by NSC23766 leads to reduced synthesis of viral proteins. (A) A549 cells, infected with A/Puerto-Rico/8/34 rec. (moi = 5), were treated with NSC23766 (100 µM) 30 min p.i.. Cell lysates were prepared at the indicated times and subjected to Western blot analysis. The viral protein synthesis was monitored by PB1, M1, and NS1 detection and visualization of ERK2 served as a loading control. (B) A549 cells were transfected with a constitutively active CMV promoter luciferase plasmid for six hours. Subsequently, NSC23766 was added at a concentration of 100 µM. Untreated cells served as a negative control, while cycloheximide treatment (10 µg/ml) was used as a positive control. After 24 h incubation, cells were subjected to luciferase assay. Data represent means ± SD of three independent experiments including three biological samples. Statistical significance was evaluated with Student's *t*-test (*** p<0.001).

To further examine the Rac1-mediated virus-supportive function during IV replication, we focused on the expression of viral proteins within the first replication cycle in the presence of NSC23766. Expression of the viral proteins PB1, M1, and NS1 was reduced upon NSC23766 treatment (Fig. 4, lanes 7 and 9) in comparison to untreated control cells (Fig. 4, lanes 6 and 8) 6 and 8 h after infection. However, to rule out a general blockage of cellular transcription or translation by NSC23766, expression of a reporter gene (luciferase) driven by a constitutive active promoter (CMV) was monitored upon NSC23766 treatment (Fig. 4B). In comparison to untreated samples, NSC23766 treatment for 24 h showed no reduction of luciferase activity, while treatment with cycloheximide, a general translation blocker, resulted in significantly lower expression of the reporter gene. Thus, it can be concluded that the inhibitor specifically reduces synthesis of IV proteins.

### NSC23766 Inhibits Viral Polymerase Activity

**Figure 5:**
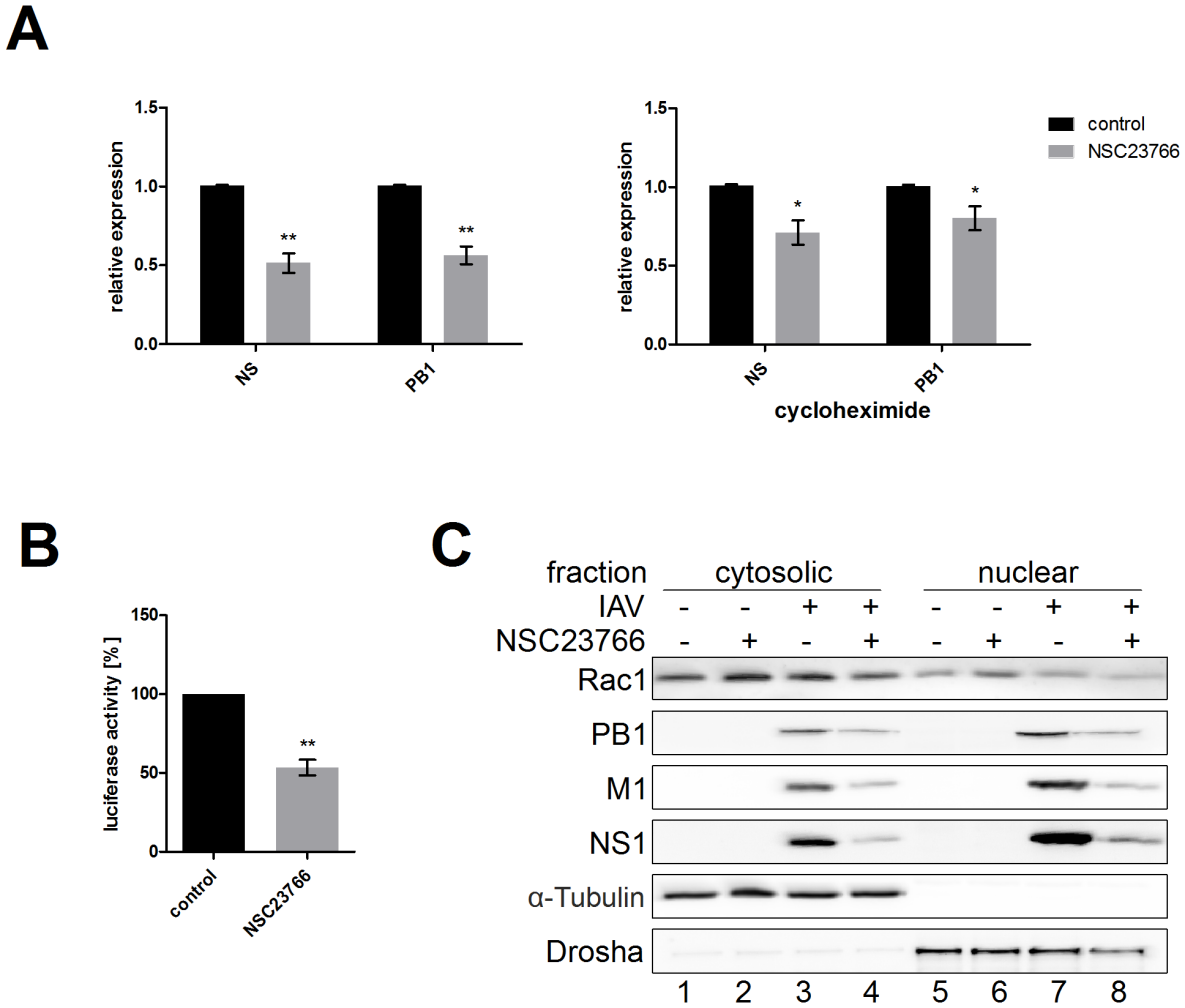
NSC23766 treatment results in reduced viral mRNA levels caused by impaired activity of the viral polymerase complex. (A) A549 cells were infected with A/Puerto-Rico/8/34 rec. (moi = 5) and subsequently treated with NSC23766 (100 µM) 30 min post-infection. Six hours p.i., the RNA was isolated for qRT-PCR and the fold increase of *ns* and *pb1* mRNA compared to uninfected cells was determined using the housekeeping gene *gapdh* as the internal standard. Treatment of cells with cycloheximide (10 µg/ml, right panel) was performed to inhibit *de novo* protein synthesis. (B) Viral polymerase activity was assayed in a mini-genome system using a luciferase reporter gene driven by the viral RNA promoter. A549 cells were transfected with plasmids encoding PB1, PB2, PA, and NP in addition to the luciferase reporter plasmid. Six hours post-transfection, NSC23766 was added at a final concentration of 100 µM and cells were incubated for a further 24 h. Luciferase activity was assayed in cell lysates. Data represent means ± SD of three independent experiments including three biological samples. Statistical significance was evaluated with Student's *t*-test (* p<0.05; ** p<0.01). (C) A549 cells were infected with A/Puerto Rico/8/34 rec. (moi = 0.01) for 30 min and were subsequently treated with NSC23766 (100 µM). After eight hours of infection nuclear extracts were prepared and subjected to Western blot analysis. The amounts of Rac1 and the viral proteins PB1, M1, and NS1 were analyzed in cytosolic and nuclear fractions. Detection of the cytosolic protein α-Tubulin and the nuclear localized protein Drosha served as control for efficient fractionation.

After demonstrating the reducing effect of NSC23766 on viral protein synthesis, we investigated if inhibitor treatment affects viral mRNA expression. Thus, the expression levels of *ns* and *pb1* mRNA in the presence and absence of the inhibitor were determined by qRT-PCR (Fig. 5A, left panel). Indeed, mRNA expression of the investigated genes was impaired upon NSC23766 treatment. Based on these results, we concluded that the reduced amount of viral mRNA expression leads to the observed lowering of viral proteins.

Nevertheless, newly synthesized viral proteins are imported into the nucleus to form additional polymerase complexes and further enhance viral gene expression. Consequently, it might also be the case that the reduced mRNA levels are the result of impaired viral protein synthesis. To rule out this possibility, we blocked *de novo* protein synthesis in virus-infected cells by adding cycloheximide and analyzed the expression of viral mRNA (Fig. 5A, right panel). In this experimental setup, only viral polymerase complexes that entered the cells with incoming virus particles are available, hence the detected amounts of viral mRNAs are completely independent of viral protein synthesis. The mRNA levels of the *ns* and the *pb1* segments were still reduced significantly and thus the observed differences in expression of *ns* and *pb1* mRNA are independent of newly synthesized viral proteins.

To test a direct impact of NSC23766 on viral polymerase activity, we performed a mini-genome assay, using a luciferase reporter gene construct under the control of viral RNA promoters [19]. The luciferase reporter plasmid was co-transfected with expression plasmids for the viral polymerase proteins (PB1, PB2, PA) and nucleoprotein (NP) 6 h prior to treatment with NSC23766. After 24 h incubation, inhibitor-treated cells showed a significantly reduced amount of luciferase activity compared to untreated control cells (Fig. 5B), indicating a lower viral polymerase activity. Taken together, Rac1 inhibition by NSC23766 leads to reduced transcription of viral genes, which is most likely the reason for the observed drop in viral protein expression and viral replication.

To directly influence the viral polymerase complex, Rac1 should be localized in the nucleus, the site of IV replication. To test if IV infection leads to increased levels of Rac1 in the nucleus and if NSC23766 might inhibit this relocalization nuclear extracts of infected A549 cells were analyzed (Fig. 5C). While Rac1 was detectable in the cytosolic as well as in the nuclear fraction, no significant changes were observable after infection or treatment with NSC23766. Nonetheless, the general presence of Rac1 in the nucleus underlines the possibility of an interaction with the viral polymerase complex in this cellular compartment. But neither a recruitment of Rac1 to the nucleus upon IV infection nor an inhibition of this relocalization by NSC23766 could be confirmed.

Furthermore, we examined the localization of the viral proteins PB1, M1, and NS1 in presence and absence of NSC23766 (Fig. 5C, lanes 3–4 and 7–8). All analyzed proteins could be detected in both fractions and their expression was reduced after treatment with NSC23766, confirming the results already presented (Fig. 4A). Since the extent of the reduction in expression of viral proteins was similar in both fractions, a specific downregulation of viral protein synthesis in the nucleus can be ruled out. This supports the conclusion that the antiviral effect of NSC23766 is independent of the nuclear import of newly synthesized viral proteins.

### Inhibition of Viral Replication by NSC23766 Shows a High Barrier for the Development of Drug Resistance

**Figure 6:**
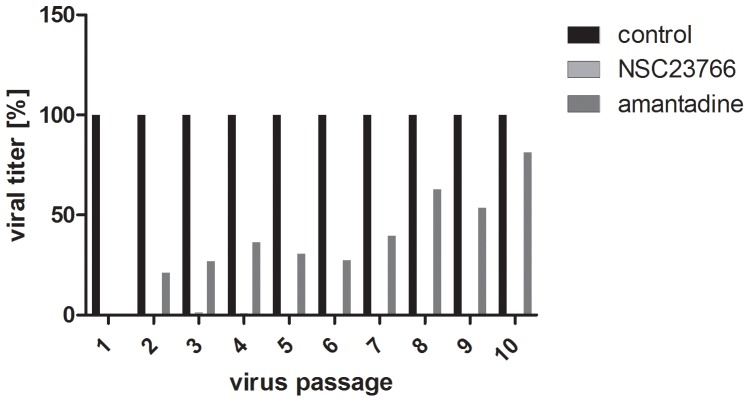
Inhibition of IV replication by NSC23766 exhibits a high barrier against the emergence of resistant virus variants. A549 cells were infected with A/FPV/Bratislava/79 (moi = 0.001) in the presence of either NSC23766 (100 µM) or amantadine (5 µM) for 24 h. Two biological samples for each treatment group were titrated, combined, and used for the next round of infection with equal amounts of virus per well. Virus titers are shown as percentage of the untreated control.

One of the most important problems with currently available drugs against IV is the frequent development of resistant virus variants against compounds directly targeting viral proteins. Therefore, we wanted to determine the potential of NSC23766 to generate resistant virus variants. A well established multi-passaging experiment under the constant evolutionary pressure of the drug was performed (Fig. 6), using the M2 ion channel blocker amantadine as positive control. After ten passages, no reduction of the antiviral effect of NSC23766 was observed, while amantadine led to rapid emergence of resistant virus variants and started to lose its antiviral effect after only two passages.

### The Interferon β Response is not Directly Inhibited by NSC23766

**Figure 7:**
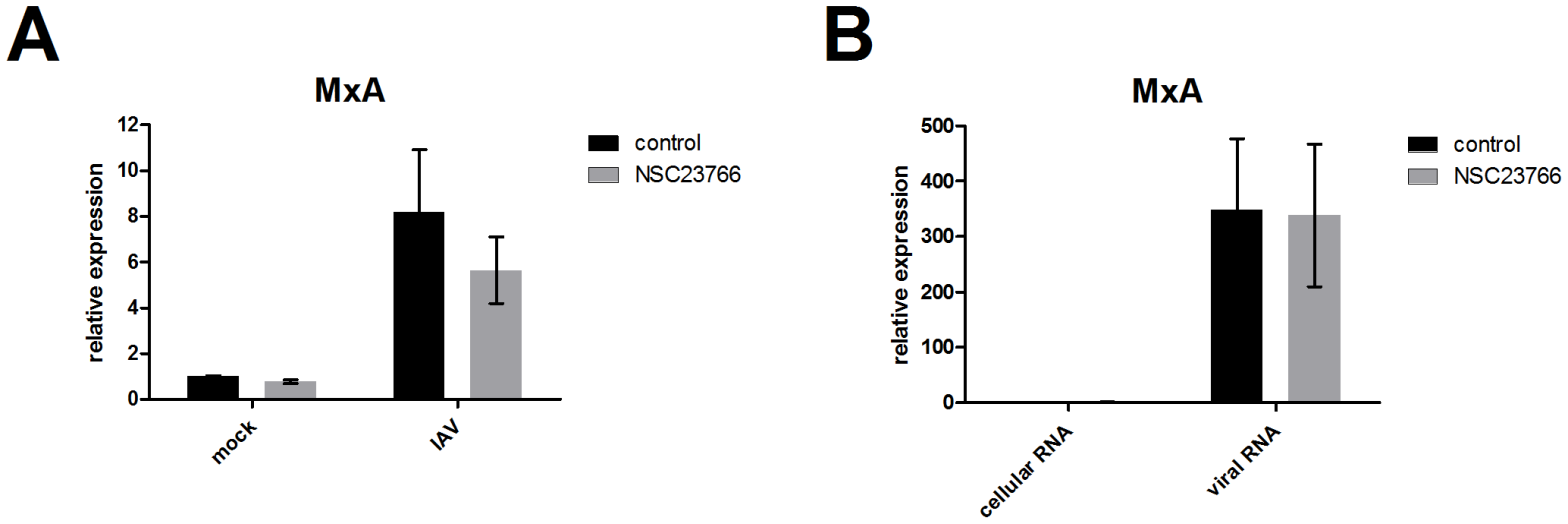
NSC23766 treatment does not directly affect expression of the interferon β regulated gene *mxa*. (A) A549 cells were infected with A/Puerto-Rico/8/34 rec. (moi = 5) and subsequently treated with NSC23766 (100 µM) 30 min after infection. (B) Alternatively, the cells were transfected with cellular or viral RNA (0.5 µg/12-well) in presence or absence of NSC23766 (100 µM). (A–B) After six hours of incubation, total RNA was isolated for qRT-PCR and the fold increase of *mxa* mRNA was determined using the housekeeping gene *gapdh* as the internal standard. Data represent means ± SD of three independent experiments including three biological samples.

So far, our results indicated a high antiviral activity of NSC23766 in IV-infected cells. This seemed to be contradictory to our former study, which identified Rac1 as positive regulator of interferon β expression after IV infections [12]. Thus, we investigated the effect of NSC23766 treatment on IV-induced interferon response. After infection of A549 cells and subsequent treatment with NSC23766, the changes in mRNA levels of *mxa*, a strictly interferon β regulated gene, were analyzed by qRT-PCR (Fig. 7A). The infection resulted in an 8-fold increase of *mxa* expression compared to mock-infected cells. The enhanced *mxa* expression was slightly reduced when Rac1 was inhibited by NSC23766 (6-fold increase compared to mock infected control). Since the NSC23766-mediated reduction in *mxa* expression might have been a secondary effect of the impaired viral replication we used viral RNA as a virus-derived non dynamic interferon stimulus in an additional experimental setting (Fig. 7B). A549 cells were transfected with RNA isolated from infected (viral RNA) or uninfected (cellular RNA) cells, in presence or absence of NSC23766. After six hours of incubation, *mxa* mRNA levels were analyzed by qRT-PCR. The transfection of viral RNA resulted in a more than 300-fold increase of *mxa* expression compared to cellular RNA-transfected control cells. The same induction of the interferon response was achieved when NSC23766 was added to the transfection medium. In summary, it can be concluded that the expression of interferon β is not directly affected by NSC23766-mediated Rac1 inhibition.

### NSC23766 Efficiently Impairs IV Replication in Mice

**Figure 8:**
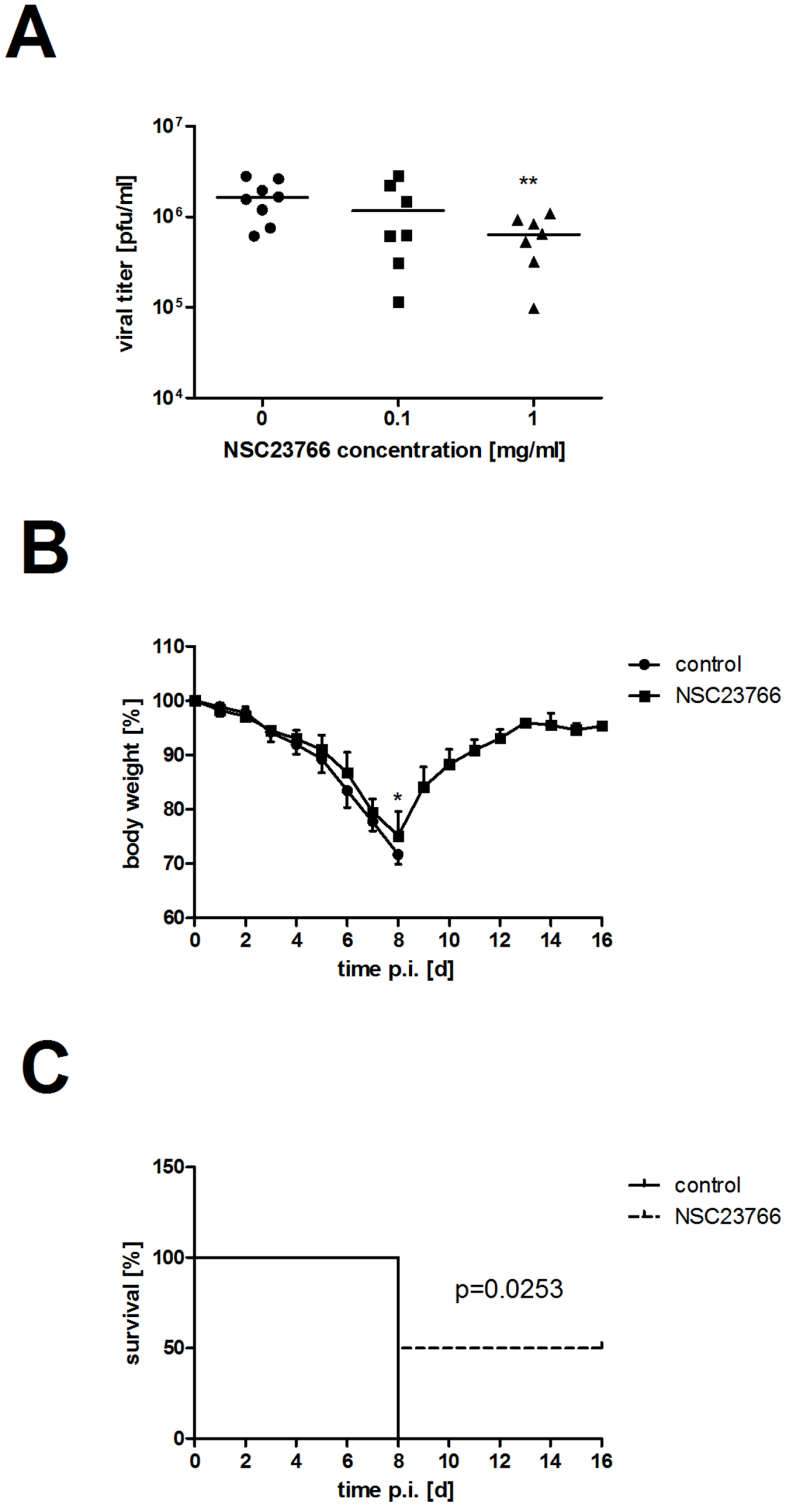
NSC23766 has an antiviral potential in mice. BALB/c mice were infected with A/Puerto-Rico/8/34 rec. (200 PFU (A) or 100 PFU (B)). Immediately after infection, treatment with nebulized NSC23766 was started (twice per day for 20 min with indicated concentrations). (A) Viral load in the lungs was determined at day three p.i. (control n = 8, 0.1 mg/ml and 1 mg/ml n = 7). (B–C) Infected mice were treated with NSC23766 (1 mg/ml) until day seven p.i and disease progression was monitored over a period of 16 days (n = 8). Mice were humanly sacrificed when they lost 25% of their body weight. (B) Mean body weight ± SD is depicted. (C) A Kaplan-Meier survival curve was plotted by GraphPad Prism 5 Software. Statistical significance was determined by Student's *t*-test (* p<0.05; ** p<0.01).

Finally, we wanted to know if NSC23766 shows antiviral activity in the mouse model. We infected BALB/c mice with 200 PFU of A/Puerto-Rico/8/34 rec. and treated them twice per day for 20 min with a nebulized NSC23766 solution (0.1 or 1 mg/ml) or water as solvent control. Three days p.i. the mice were sacrificed and the viral loads in the lungs were analyzed (Fig. 8A). In comparison to untreated mice, the replication of IV in NSC23766-treated mice was reduced. While the treatment with 0.1 mg/ml resulted in a moderate drop of viral titers (29%), the concentration of 1 mg/ml was sufficient to significantly reduce viral lung loads by 61%.

These promising results led us to the question if treatment with NSC23766 can diminish disease progression and prolong the survival of infected mice. Therefore, BALB/c mice were treated twice per day with NSC23766 (1 mg/ml) or water as solvent control for eight consecutive days beginning immediately after infection with A/Puerto-Rico/8/34 rec. (100 PFU). The body weight was documented for 16 days as an objective readout for disease progression (Fig. 8B). Both treatment groups lost body weight, which was slightly attenuated in the inhibitor-treated group from day four after infection. The difference in mean body weight loss became significant at day eight p.i. when it reached 3.5%. While all eight solvent-treated mice had to be euthanatized at day eight post infection, four out of eight inhibitor-treated mice survived the infection and recovered completely (Fig. 8B). These data were used to draw a Kaplan-Meier survival curve, to illustrate the prolonged survival of NSC23766-treated mice (Fig. 8C). Taken together, the attenuation of viral replication and the prolonged survival by NSC23766 treatment in mice underline the critical role of Rac1 *in vivo*.

## Discussion

Infections with IV are still a serious threat for mankind and the constant emergence of new virus strains are a challenging task for global healthcare. In addition, the frequent development of resistance against currently available drugs underlines the urgent need for new antiviral strategies. Here we report the antiviral potential of the specific Rac1 inhibitor NSC23766 against a wide variety of IVs in cell culture and in the mouse model. Furthermore, we present first evidence for an involvement of Rac1-dependent signaling in viral polymerase activity.

We had shown before that Rac1 is involved in the onset of the type-I interferon response upon IV infections and that expression of dominant-negative mutants results in increased virus titers [12]. At first glance this might appear to be in contrast to the reduced viral titers after treatment with NSC23766 (Fig. 1) or transfection of Rac1 and Tiam1 siRNA (Fig. 3). This discrepancy might be explained by spatio-temporal patterns of the cellular factors or mechanistic differences in the mode of inhibition.

There is accumulating evidence that one enzyme is often able to fulfill antiviral as well as virus-supportive functions during viral replication in a time- and probably location-dependent manner [13]. Such a phenomenon was also observed in the case of JNK inhibition, when expression of dominant-negative mutants led to increased virus titers while chemical inhibition resulted in reduced viral replication [25], [26]. Thus, different branches of the signaling pathway might be inhibited separately by diverse methods [26]. In the present case, the high specificity of NSC23766, which targets only the Rac1 activation by Tiam1 and Trio, might explain the discrepancy in the effect on viral replication in comparison to the general block of Rac1 by dominant-negative mutants. Thus the different modes of action of both applied inhibitory procedures are most likely the reason for the different outcomes. While NSC23766 interferes with a specific subset of activating factors, namely Tiam1 and Trio, the dominant negative mutant competes with the endogenous Rac1 for various cellular interaction partners. In consequence, different effects on Rac1-mediated signaling are not surprising.

Rac1 signaling was shown to be involved in the entry process of a number of enveloped viruses including human immunodeficiency virus, vaccinia virus, and African swine fever virus [5]-[7]. Surprisingly, a recent publication showed that endocytosis of IV does not depend on Rac1 signaling [16]. Thus, we were prompted to search for the mechanism of the observed antiviral activity of NSC23766 post entry. We could show that upon Rac1 inhibition by NSC23766, viral polymerase activity is reduced (Fig. 5B), which seems to result in lowered viral mRNA levels (Fig. 5A) and subsequently impaired synthesis of viral proteins (Fig. 4A).

The transcription and replication of the IV genome takes place in the nucleus, suggesting that Rac1 might be needed at the same place to support viral propagation. Although Rac1 is mainly located in the cytoplasm, a growing number of recent publications suggest additional localization and functions in the nucleus. Rac1 contains a functional nuclear localization signal [27] and can enter the nucleus in its activated form by interaction with the nuclear import receptor karyopherin α2 [28]. Furthermore, Rac1 is necessary for the nuclear import of transcription factors of the STAT family, where it is suggested to function as a nuclear transport chaperone [29]. Here we could detect Rac1 in the nucleus of A549 cells (Fig. 5C). Due to these results an direct interaction with the viral polymerase complex during replication seems to be possible. However, we did not observe significant changes in Rac1 localization after IV infection or NSC23766 treatment. In consequence, effects on Rac1 localization can be ruled out as the mechanism for NSC23766-mediated inhibition of IV replication. Furthermore, the viral proteins were reduced to the same extent in the cytosol and the nucleus (Fig. 5C), excluding Rac1 as nuclear import factor for newly synthesized IV proteins.

We have shown that NSC23766 efficiently impairs replication of a wide variety of different influenza viruses in cell culture (Fig. 1C) and that treatment with the effective dose does not lead to unspecific cell cytotoxicity (Fig. 2). Together with the significant reduction of viral propagation in mice (Fig. 8A) and the prolonged survival rate of infected mice (Fig. 8C), inhibition of Rac1 by NSC23766 treatment seems to be a promising target for anti-IV intervention. Further development and modification of Rac1 inhibitors might offer even more potent drugs for anti-influenza therapy by targeting Rac1 [30].
